# Experimental and Computational Studies of Compression and Deformation Behavior of Hafnium Diboride to 208 GPa

**DOI:** 10.3390/ma15082762

**Published:** 2022-04-09

**Authors:** Kaleb Burrage, Chia-Min Lin, Cheng-Chien Chen, Yogesh K. Vohra

**Affiliations:** 1 Neutron Sciences Directorate, Oak Ridge National Laboratory, Oak Ridge, TN 37830, USA; burragekc@ornl.gov; 2 Department of Physics, University of Alabama at Birmingham, Birmingham, AL 35294, USA; lincm@uab.edu

**Keywords:** transition metal borides, high pressure, diamond anvil cell, equation of state, shear strength

## Abstract

The compression behavior of the hexagonal AlB_2_ phase of Hafnium Diboride (HfB_2_) was studied in a diamond anvil cell to a pressure of 208 GPa by axial X-ray diffraction employing platinum as an internal pressure standard. The deformation behavior of HfB_2_ was studied by radial X-ray diffraction technique to 50 GPa, which allows for measurement of maximum differential stress or compressive yield strength at high pressures. The hydrostatic compression curve deduced from radial X-ray diffraction measurements yielded an ambient-pressure volume V_0_ = 29.73 Å3/atom and a bulk modulus K_0_ = 282 GPa. Density functional theory calculations showed ambient-pressure volume V_0_ = 29.84 Å3/atom and bulk modulus K_0_ = 262 GPa, which are in good agreement with the hydrostatic experimental values. The measured compressive yield strength approaches 3% of the shear modulus at a pressure of 50 GPa. The theoretical strain-stress calculation shows a maximum shear stress τ_max_~39 GPa along the (1−10) [110] direction of the hexagonal lattice of HfB_2_, which thereby can be an incompressible high strength material for extreme-environment applications.

## 1. Introduction

Hafnium diboride (HfB_2_) belongs to a class of AlB_2_-type transition metal borides that have exhibited superb physical and thermal properties. The high bulk modulus (K_0_ = 260–350 GPa [1,2]), low thermal expansion coefficient (7.49 × 10^−6^ K^−1^) [3], and high shear to bulk modulus ratio (G_0_~270 GPa) have brought much attention to HfB_2_ for applications as a material exposed to extreme environments. The combination of strength and temperature resilience has garnered attention from the field of aerospace engineering for hypersonic and re-entry vehicles into Earth’s atmosphere. HfB_2_ is special among the refractory metal borides for the applications listed as it has a lower density (10.5 g/cm^3^) [4] than other ultra-incompressible metal borides such as ReB_2_ (12.7 g/cm^3^) and WB_2_ (10.8 g/cm^3^) while still retaining similar elastic moduli values [4]. The incorporation of boron into the hexagonal crystal lattice (symmetry group P6/mmm) also enhances neutron absorption capabilities for protection from damaging radiation that can embrittle materials. Relatively recent theoretical studies have shown that these refractory transition metal borides are as efficient as water at moderating neutrons and suggest applications in reactor and future fusion devices [5]. The broad range of application of HfB_2_ for industrial tool use and other extreme environments has led researchers to study its compression behavior, though only to relatively modest pressures 30–50 GPa compared to the conventional limits of commonly used diamond anvil cells (DAC) that can reach >200 GPa. The compression behavior or equation of state for HfB_2_ is currently not available in the ultrahigh-pressure region despite the propensity for structural and electronic transitions of Group IV metals with narrow *d*-bands and broad *sp*-bands to occur with high pressure [6]. There is also an interest in studying how different pressure states (whether nonhydrostatic or hydrostatic) affect compression behavior. Some previous studies have used pressure mediums such as noble gases to make the sample compression in a DAC more hydrostatic, but this is best classified as quasi-hydrostatic due to crystallization of the gas or fluid with pressure and limited in the applied pressure range (~30 GPa) [7,8]. Theoretically, *ab initio* evolutionary algorithms have indicated that the AlB_2_ structure with the transition metal and boron atoms sitting at the origin and the (1/3, 2/3, 1/2) Wyckoff sites can persist to a few hundred GPa in certain diborides such as ZrB_2_ [9]. However, whether the structure in ZrB_2_ or HfB_2_ can be stable at such as high pressure remains experimentally unknown.

In this study, HfB_2_ is compressed using beveled diamond anvils to 208 GPa to probe volumetric compression in a nonhydrostatic pressure environment, accomplished by an axial X-ray diffraction technique. This is also combined with a separate study using DAC with radial X-ray diffraction (R-XRD) and lattice strain theory (LST) to determine the hydrostatic pressure volume relation, as well as an estimation of its compressive yield strength. Density functional theory (DFT) calculations are also performed, and the theoretical results show good agreement with the experimental results on compression and deformation behavior.

## 2. Materials and Methods

The nonhydrostatic compression study on HfB_2_ was conducted using a pair of beveled diamond anvils with a central flat of 30-microns, 8-degree bevel to 300-micron outer diameter. A spring steel gasket was indented to 30-micron thickness and laser drilled for a sample hole of 5-microns for sample placement. The HfB_2_ sample was in powder form (Alfa Aesar, 99.5% metals basis) and mixed in a 3:1 ratio with platinum powder (Alfa Aesar, 99.97% purity) that was used for pressure calibration. Platinum was chosen as the pressure calibrant as its XRD peaks will not overlap with the sample, while the 3:1 ratio was chosen to ensure the platinum intensity does not overwhelm that of HfB_2_ for later structural refinement. Given that platinum has a similar shear modulus to the base metal in HfB2, it is not expected that platinum will alter shear strength measurements significantly. This is later addressed in the Discussion section. The axial X-ray diffraction was conducted at the High-Pressure Collaborative Access Team (HPCAT) at Beamline 16-ID-B, Advanced Photon Source, Argonne National Laboratory.

For determination of hydrostatic components of compression, radial X-ray diffraction at beamline 16-BM-D, APS, Argonne National Laboratory was conducted. A pair of 300-micron flat diamond anvils were used for HfB_2_ sample compression. The same HfB_2_ and Pt sample mixture from the nonhydrostatic compression study was used for the R-XRD study and was placed in a beryllium gasket on the diamond culet with a sample hole of 70 microns in diameter. The X-ray beam was focused through Double Multilayer Monochromator into a collimating pinhole to a beam size of 3.4 µm (vertical) × 4.7 µm (horizontal) at 30 keV.

The pressure state at the center of a sample in a DAC is defined as nonhydrostatic due to differential stress between the axial and radial directions. The maximum stress lies along the diamond compression axis, while the minimum stress is exerted radially by the gasket material. This differential stress is a measure of compressive yield strength of the sample giving rise to a yielding of the crystallites that can be observed experimentally via R-XRD. To ensure the lattice strain is observed in the R-XRD profiles, a nonhydrostatic pressure state is necessitated, and no pressure medium is used in the sample hole. The lattice strain can be shown using Lattice Strain Theory (LST) produced by Singh et al. [10], where lattice strain from the nonhydrostatic environment will deform the measured d-spacing d_m_(hkl) by:

d_m_(hkl) = d_p_(hkl) [1 + (1 − 3cos^2^ψ)Q(hkl)], (1)

where d_p_(hkl) is the hydrostatic component of the d-spacing, ψ is the angle between the cell compression axis and normal to the diffracting plane, and Q(hkl) is the lattice strain. Experimentally, the angle ψ can be determined by the relation ψ = cos(δ)cos(θ), where *δ* is the azimuthal angle around the XRD image and θ is the diffracting angle. The angle ψ can then be chosen such that 1 − 3cos^2^(ψ) = 0, in which case the d-spacing measured at this angle is the hydrostatic d-spacing d_m_(hkl) = d_p_(hkl). This provides a unique method for determining hydrostatic components of compression while the sample is under nonhydrostatic stress.

The lattice strain term Q(hkl) is also very useful as it can be utilized to derive the differential stress and shear strength of a sample. This is shown in Equation (2):

Q(hkl) = (*t*/3){α[2 G_R_(hkl)] − 1+(1 − α)[2 G_V_] − 1)}, (2)

where *t* is the differential stress, α is a weighted factor between 0 and 1, and G_R_(hkl) and G_V_ are the shear moduli approximations for the Reuss (iso-stress) and Voigt (iso-strain) conditions. By assuming the Voigt approximation, Equation (2) can be simplified to form a lower bound to compute the differential stress (*t*) and shear strength (τ):

*t* = 6G <Q(hkl)> = 2τ. (3)

The measured d-spacing d_m_(hkl) from Equation (1) and the lattice strain term Q(hkl) were determined in this study by using the Rietveld refinement software MAUD [11]. The collected R-XRD images were integrated into azimuthal sections of δ = 5 degrees to determine the angle ψ, and each section was sequentially refined in MAUD to determine the variation of 1 − 3cos^2^(ψ) with d_m_(hkl), which allowed for the calculation of d_p_(hkl) for both HfB_2_ sample and Pt pressure marker. The hydrostatic d-spacing d_p_(hkl) for Pt as well as the d-spacing for the nonhydrostatic experiment to 208 GPa were used in the Birch–Murnaghan equation of state (BM EoS) to determine pressure using Pt equation of state parameters derived by Yokoo et al. [12]:

P(V) = (3/2) K_0_ [x^7/3^ − x^5/3^] [1 + 0.75 (K_0′_ − 4) x^2/3^ − 1)], with x = V_0_/V. (4)

The first-principles calculations were performed using VASP (the Vienna Ab initio Simulation Package, version 5.4.4) [13,14], which is a plane-wave pseudopotential density functional theory (DFT) code [15,16]. The Perdew–Burke–Ernzerhof generalized gradient approximation (GGA-PBE) [17] functional and the projector augmented wave (PAW) method [18,19] were employed in our calculations. A plane-wave basis set with an energy cut-off of 600 eV and a fine 33 × 33 × 33 (resolution = 0.01 × 2π/Å) Monkhorst-Pack k-point grid were chosen. Structural relaxation calculations were performed with convergence criteria of 10^−3^ eV/Å and 10^−6^ eV/cell for the atomic force and self-consistent electronic steps, respectively. After the structure optimization, we computed the elastic tensor by the strain-stress method [13,14,20,21] in VASP. The bulk modulus K and shear modulus G are derived from the elastic constants using the Voigt–Reuss–Hill approximation [22,23,24], which averages the upper and lower bounds of the moduli.

**Figure 1:**
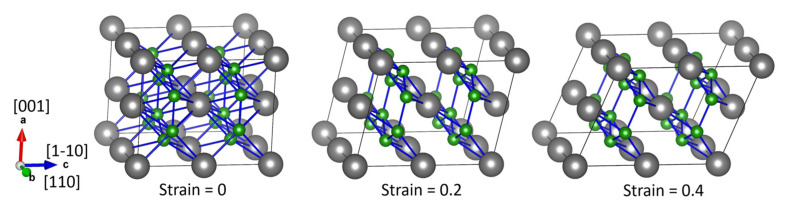
Schematic of shear deformation in HfB_2_ along the (001) [1−10] shear direction at strain values 0, 0.2, and 0.4, respectively. The gray and green balls represent the Hf and B atoms, respectively.

We also computed the tensile and shear strengths of HfB_2_ by using the QE (Quantum ESPRESSO, version 6.3) [25,26,27] DFT package. The PAW method and GGA-PBE functional were utilized as well. The same calculation settings and convergence criteria as those in the VASP calculations were considered. We used a fully relaxed crystal structure to construct a 2 × 2 × 2 supercell for computing the strain-stress curves. A k-grid of 11 × 9 × 9 points was used in the supercell calculations. Tensile strains were applied along the [001], [100], [110], and [111] directions, respectively, with a strain value up to 0.4 in a strain step of 0.01. In each step, the lattice constants and atomic positions of the tensile axis were fixed, while the lattice constants and atomic positions of the other two axes were allowed to relax fully. The shear strain-stress curves were obtained in a similar way by considering shear deformation in different directions on various planes. Figure 1 shows an example of shear deformation calculation on the (001) plane along the [1−10] direction with different strain values. In each step of the shear deformation calculation, we fix the lattice constant and atomic positions of the axis perpendicular to the shear plane (a-axis in Figure 1) and completely relax the lattice constants and atomic positions of the other two axes parallel to the shear plane (b and c axes in Figure 1). The structures were visualized by the VESTA software (version 3.4.8) [28].

Since the atoms are located at high-symmetry Wyckoff positions in HfB_2_, the actual atomic positions along the strain direction will be directly determined by the lattice parameters. We have benchmarked our QE calculations by comparing to results in the literature for ReB_2_ [29] and Mg_2_Si [30] using VASP, in which cases the applied strain is fixed, while the other five independent components of the strain tensors and all the atom positions were simultaneously relaxed. The critical strain and maximal stress values determined from these different approaches and software agree at a quantitative level.

## 3. Results

**Figure 2:**
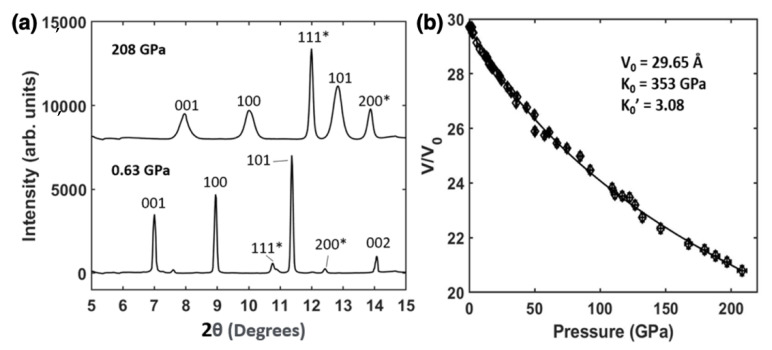
(**a**) Axial X-ray diffraction patterns of HfB2 and Pt (*) powder at 0.63 GPa and 208 GPa. (**b**) Pressure volume curve of HfB_2_ to 208 GPa with the fitted 3rd Order Birch–Murnaghan equation of state. The fitted parameters are shown in the inset.

The hexagonal AlB_2_ phase of the HfB_2_ sample was found to be stable to the highest pressure of 208 GPa (V/V_0_ = 0.7). Figure 2a shows the sample HfB2 and Pt mixture at 0.63 GPa and 208 GPa, while Figure 2b shows the nonhydrostatic compression of HfB_2_ reaching a maximum pressure of 208 GPa fitted with the 3rd Order BM EoS. The least-square fitting of HfB_2_ pressure-volume data shows a bulk modulus K_0_ = 353 GPa with first pressure derivative K_0′_ = 3.08 and an initial volume V_0_ = 29.65 Å.

**Figure 3:**
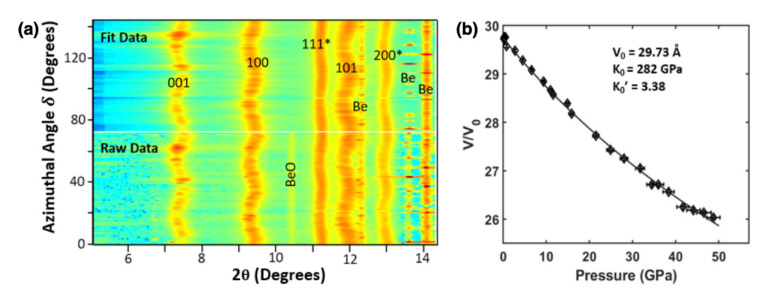
(**a**) R-XRD image of raw and Rietveld refined fits of HfB2 and Pt (*). Beryllium gasket peaks are labelled with beryllium oxide impurity. (**b**) Hydrostatic pressure volume curve of HfB_2_ to 50 GPa with the fitted 3rd Order Birch–Murnaghan equation of state. The fitted parameters are shown in the inset.

The hydrostatic pressure-volume curve derived from LST and R-XRD is displayed in Figure 3 with the corresponding 3rd Order BM EoS fit and derived elastic parameters. The initial volume from R-XRD hydrostatic parameters gives a slightly higher volume V_0_ = 29.74 Å with a lower bulk modulus K_0_ = 282 GPa (with first pressure derivative K_0′_ = 3.38) compared to the nonhydrostatic case. This decrease in bulk modulus for more hydrostatic environments is also noted in other literature for HfB_2_ with similar moduli values [1,2]. This is attributed to lattice strain being underestimated under nonhydrostatic stress in axial diffraction geometry [10], i.e., the measured volume for nonhydrostatic cases can be larger with increasing pressure (and strain), rendering a seemingly higher bulk modulus. For HfB_2_, this effect seems to be profound as the difference in nonhydrostatic and hydrostatic bulk moduli are ~82 GPa for this study and ~79 GPa in Laing et al. for quasi-hydrostatic bulk modulus [1]. This will be discussed further later in the Discussion section.

We next present DFT-GGA calculation results, which can be compared to HfB_2_ experiments under hydrostatic pressure. The calculated lattice constants, volume, bulk, and shear moduli under ambient conditions are a_0_ = 3.14 Å, c_0_ = 3.49 Å, V_0_ = 29.84 Å^3^/atom, K_0_ = 262 GPa, and G_0_ = 246 GPa, respectively. These calculated values are in good agreement with our experiments (V_0_ = 29.73 Å^3^/atom, K_0_ = 282 GPa) as well as other theoretical and experimental values (V_0_ = 29.78–29.90 Å^3^/atom, K_0_ = 261–395 GPa, G_0_ = 227–248 GPa) reported in the literature [1,2,31,32,33,34].

**Figure 4:**
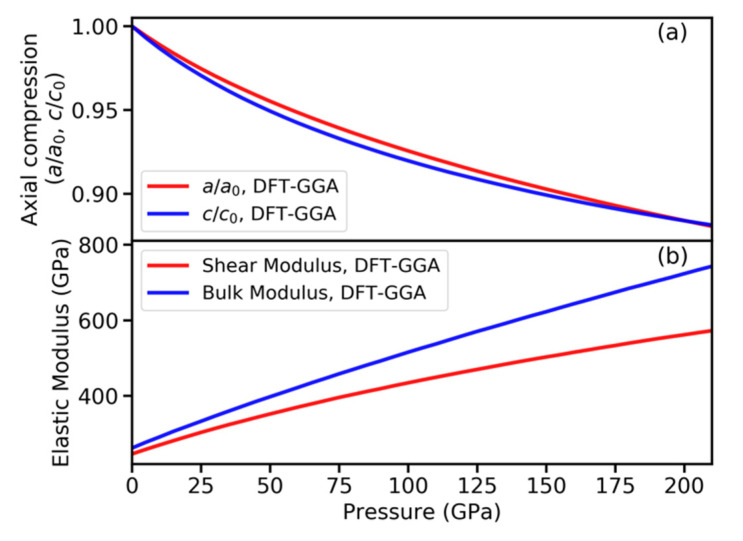
Pressure dependences of (**a**) axial compression of lattice parameters a/a_0_ and c/c_0_, and (**b**) bulk and shear moduli for HfB_2_. The density functional theory (DFT) calculations used the generalized gradient approximation (GGA) functional.

Figure 4a shows the calculated hydrostatic axial compression ratios a/a_0_ and c/c_0_ up to 210 GPa. The results indicate a slight anisotropic compression behavior of HfB_2_. The c-axis compresses more easily than the a-axis, and the compression anisotropy decreases gradually with increasing pressure up to 125 GPa. Figure 4b shows the variation of bulk and shear moduli with pressure. The high bulk and shear modulus values under compression suggest highly incompressible and deformation-resistant behavior of HfB_2_.

**Figure 5:**
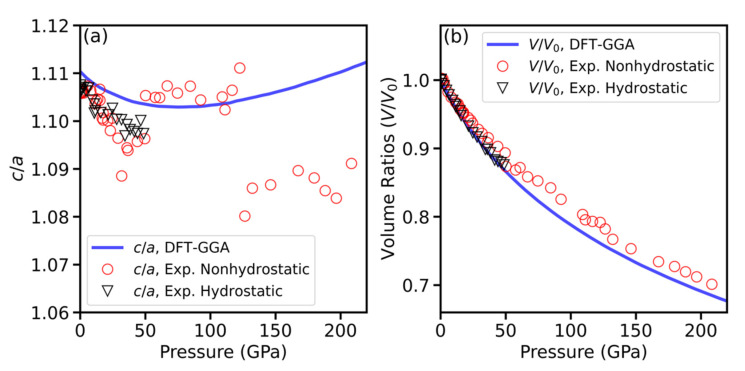
Pressure dependences of (**a**) lattice parameter c/a ratio, and (**b**) unit cell volume V/V_0_ ratio for HfB_2_. The density functional theory (DFT) calculations used the generalized gradient approximation (GGA) functional and are plotted together with the experimental data.

The calculated c/a ratio of HfB_2_ under pressure is plotted in Figure 5a along with the experimental data, including both nonhydrostatic and hydrostatic results. The measured c/a ratio decreases with increasing pressure. The anisotropy between the c and a-axes is noted for the pressure region 0–50 GPa, with the c-axis being the most compressible. Figure 5b shows the calculated volume compression V/V_0_ under pressure along with the experimental values. Overall, the calculated results are in good agreement with the experiments up to 208 GPa. It is noted that the c/a ratio appears to decrease slightly with pressure in the low-pressure regime, and it starts to increase with pressure in the higher-pressure range.

**Figure 6:**
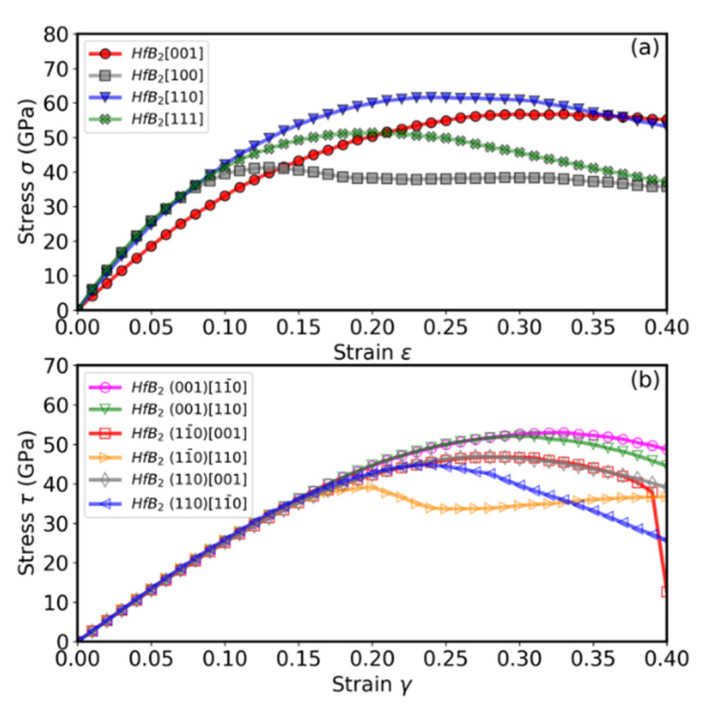
Theoretical stress-strain curves of HfB_2_ under (**a**) tensile deformation, and (**b**) shear deformation, up to strain value of 0.4 with a strain step of 0.1 along various directions.

Figure 6 shows the calculated shear and tensile stress-strain relationships for HfB_2_. The strain-stress curves show a linear behavior at small strain values (Hooke’s law). At larger strains, a nonlinear behavior follows. The critical value is reached when the stress starts to decrease with strain. When the critical point is exceeded, the structure can become unstable, which leads to deformation or fracture. The maximum stress determines the upper limit of a material’s mechanical strength.

Figure 6a shows the calculated tensile stress as a function of strain along the [001], [100], [110], and [111] directions. The peak tensile stress in the [100] direction corresponds to the ideal tensile strength of HfB_2_ because it has the lowest value among the four directions. The ideal tensile stress is ~41.31 GPa at a critical strain value of 0.13. Likewise, ideal shear strength occurs at the lowest peak shear stress in all directions. Figure 6b shows that the ideal shear strength of HfB_2_ is in the (1−10) plane along the [110] direction. The ideal shear stress is ~39.16 GPa at a critical strain value of 0.2. It is noted that the theoretical shear strength of a perfect crystal is approximately equal to the shear modulus G_0_ divided by 2π. Our calculation results are consistent with this theoretical estimation. Table 1 lists the calculated data for the maximum tensile and shear stresses in each direction and their corresponding critical strain values.

## 4. Discussion

**Figure 7:**
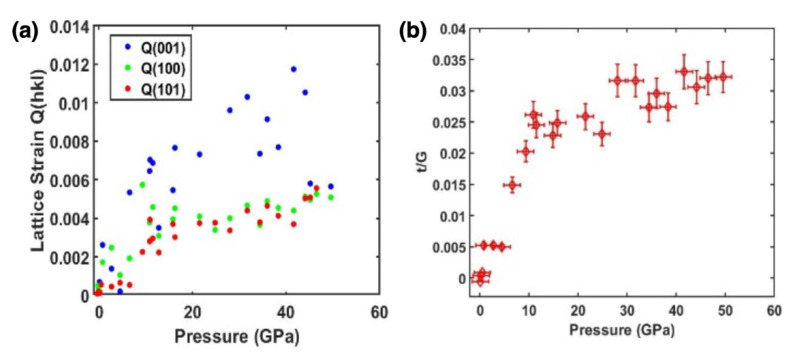
(**a**) Orientation dependent lattice strain Q(hkl) in HfB_2_ to 50 GPa. (**b**) The differential stress normalized to shear modulus (*t*/G ratio) for HfB_2_ as a function of pressure to 50 GPa.

HfB_2_ is similar in crystal structure to other AlB_2_-type transition-metal borides (TMBs) such as TiB_2_ and ZrB_2_, and has certain similarities to hexagonal WB_2_-type TMBs. These materials typically share similar elastic properties, with nonhydrostatic bulk modulus values in the range of 260–400 GPa (e.g., K_0_ = 263–395 GPa for HfB_2_ [1,2], K_0_ = 350–366 GPa for ReB_2_ [35,36,37,38], and K_0_ = 358 GPa for Os_2_B_3_ [39,40]). However, it is interesting to note the behavior of HfB_2_ with respect to differing pressure (hydrostatic versus nonhydrostatic) environments as compared to other TMBs. ReB_2_ has displayed a nonhydrostatic bulk modulus of 360 GPa and a hydrostatic bulk modulus of 366 GPa. This is further replicated by DFT calculations (K_0_ = 360 GPa) and quasi-hydrostatic compression to 30 GPa (K_0_ = 360 GPa) [35,36,37,38]. HfB_2_, however, has shown a ~ 80 GPa difference in bulk modulus between our nonhydrostatic and hydrostatic experiments, as well as in nonhydrostatic and quasi-hydrostatic studies in the literature [1,2]. This suggests the impact of high compressive yield strength of HfB_2_ on the nonhydrostatic X-ray diffraction measurements. To further illustrate this point, we have analyzed the maximum differential stress (*t*) obtained from the radial X-ray diffraction data using Equation (3) and average measured lattice strains <Q(hkl)>. Figure 7 shows that the maximum differential stress (*t*) or compressive yield strength normalized to shear modulus (G) increases with increasing pressure and approaches a limiting value of 3% of the shear modulus (0.03 G).

The lattice strains Q(hkl) for (001), (101), and (002) planes given by Equation (2) for HfB_2_ as a function of pressure are also shown in Figure 7. The (001) plane shows a considerably higher strain Q and thus higher compressive yield strength than the other planes. This is an interesting result, as the axis of most incompressibility is along the a lattice parameter while maximum strength is along the (001) plane. This is actually opposite to what is seen in WB_2_-type TMBs, which display higher incompressibility along the c-axis and maximum strength in the (100) plane [36,37]. The WB_2_-type differs from the AlB_2_-type in that the interstitial boron layers are puckered, whereas the AlB_2_ displays a flat plane of boron atoms [41,42]. In a puckered boron layer, the distance between TM and B atoms is shorter than that of the flat boron layer. Therefore, the puckered boron layer has strong covalent hybridization between TM and B atoms. It has been shown in some WB_2_-type structures that electron density congregates along the c-axis, giving them their characteristic incompressibility [40]. Based on results in the c/a ratio and the lattice-dependent strain Q(hkl) in this study, it is inferred that the orientation of boron atoms in the AlB_2_-type structure of HfB_2_ plays a large role in the selection of electronic density along the a-axis as compared to the WB_2_-type structure. When the applied pressure increases, the Hf-Hf bond along the c-axis becomes shorter, which leads to a more repulsive electron-electron interaction and a stronger hybridization between Hf and B atoms; therefore, the compression of the c-axis can then become comparable to that of the a-axis at higher pressure.

Finally, our DFT calculations show that the weakest tensile strength is along the [100] direction, with a peak tensile stress value of σ~41.3 GPa at critical strain ε~0.13. Moreover, the ideal shear strength of HfB_2_ occurs along the (1–10) [110] direction, with a peak shear stress value of τ~39.2 GPa at critical strain ε~0.2. Based on the Frenkel model [43], the shear stress τ can be approximated as a sinusoidal stress-strain relationship:

τ= [Ga/2πh] sin(2πx/a),(5)

where G is the shear modulus, a is the spacing between atoms in the direction of shear stress, h is the atomic row spacing, and x is the shear translation. The maximum shear stress value τ_max_ can be obtained by letting sin(2πx/a) = 1. Since h~a, τ_max_~G/2π~0.16 G, our DFT-calculated shear modulus value of 246.5 GPa will lead to τ_max_ = 246.5 GPa/2π ≈ 39 GPa, which is consistent with the ideal shear strength from the strain-stress curves. Experimentally, the compressive yield strength of 0.03 G is smaller, potentially due to crystal imperfections. The presence of platinum (an elastically weaker material than HfB_2_) within the sample hole of the DAC poses a natural question to what degree the mechanical properties of HfB_2_ are altered. Given the excellent agreement in shear modulus, bulk modulus, and lattice parameter in this study with other literature values, it is not expected that the platinum pressure calibrant would cause the large difference in theoretical and experimental shear strength values of HfB_2_. A further study into fully sintered HfB_2_ without the presence of platinum would have to be conducted to understand this effect fully.

## 5. Conclusions

The equation of state of HfB_2_ was measured up to 208 GPa in a diamond anvil cell employing platinum as an internal pressure standard. The hexagonal AlB_2_ was found to be stable up to 30% volume compression or V/V_0_ = 0.7. The c-axis was observed to be more compressible relative to the a-axis below 50 GPa, in agreement with theoretical calculations. The hydrostatic compression curve deduced from radial X-ray diffraction measurements yielded an ambient-pressure volume V_0_ = 29.73 Å^3^/atom and a bulk modulus K_0_ = 282 GPa. Density functional theory calculations showed ambient-pressure volume V_0_ = 29.84 Å^3^/atom and bulk modulus K_0_ = 262 GPa, which are in good agreement with the hydrostatic experimental values. The theoretical strain-stress calculation shows that the weakest deformation direction is along the (1–10) [110] shear direction, and the maximum shear stress is τ_max_~39 GPa~G_0_/2π. Our comprehensive experimental and computational studies of the compression and deformation behavior of HfB_2_ provide new results and understanding of this incompressible high strength material, which has potential applications in extreme environments.

## Figures and Tables

**Table 1:** Maximum tensile σ_max_ and shear stresses τ_max_ (in GPa) and their corresponding critical strain ε_max_ values along different directions. The results are based on density functional theory (DFT) calculations using the generalized gradient approximation (GGA) functional.

Tensile Deformation			Shear Deformation		
	σ_max_	ε_max_		τ_max_	ε_max_
[001]	56.84	0.33	(001) [110]	52.06	0.30
[100]	41.31	0.13	(001) [1−10]	53.01	0.33
[110]	61.59	0.24	(110) [001]	46.94	0.27
[111]	51.32	0.20	(110) [1−10]	44.82	0.24
			(1−10) [001]	46.78	0.29
			(1−10) [110]	39.16	0.20

## Data Availability

The experimental and computational data presented in this study are available upon request to the corresponding authors.
